# 40 years of editorials

**DOI:** 10.1590/0102-311XEN025924

**Published:** 2024-03-11

**Authors:** Marilia Sá Carvalho, Luciana Dias de Lima, Luciana Correia Alves

**Affiliations:** 1 Programa de Computação Científica, Fundação Oswaldo Cruz, Rio de Janeiro, Brasil.; 2 Escola Nacional de Saúde Pública Sergio Arouca, Fundação Oswaldo Cruz, Rio de Janeiro, Brasil.; 3 Instituto de Filosofia e Ciências Humanas, Universidade Estadual de Campinas, Campinas, Brasil.

Editorials have different objectives in a scientific journal. Some highlight a specific article from an issue, which are called “Editor’s Choice”. Others address important aspects of the editorial policy that help authors choose the journal to submit their articles, mainly providing an answer to a common question authors have: “Is my article really suitable for the CSP?” Editorials also show the opinion of the editorial board on aspects of the scientific work in the journal field - in the case of the CSP, Public Health, and its view and political positioning regarding relevant topics in the context of science, society, and health.

In fact, these topics are often considered “outside” from what the magazine should publish, as if science were neutral and had to be out of the political debate. But it is not! Even the most renowned scientific journals in the world assume positions on current and effervescent issues, as in this editorial in traditional *The Lancet* ^1^: *Gaza’s Crisis Must Not Be Overshadowed*, published in 2006. It also happens when the journals assume a clearly critical position presented in the form of declared support to electoral candidates that fight against denial of science and the dismantling of public policies and essential services for the population ^2^. Democracy is a central theme for the CSP, and we seek to speak up in critical moments of threats to democracy and the right to health ^3^ ^,^ ^4^.

Reflections on the journal evolution, published on its 10th, 15th, 20th, 25th, and 30th anniversaries, and the ones we provide now in this 40th issue, highlight a necessary discussion. After all, a journal does not survive only with the submission and publication of manuscripts, but also with choices and priorities for publication, defined by the editorial board according to the context.

In its launch editorial, the CSP stated its commitment, which has been the same since then: “*...open to the collaboration of professionals from any national or foreign institutions, it proposes to be a permanent forum for debate on issues that are directly or indirectly linked with Public Health* (...) *thus participating in the dissemination and circulation of ideas that will contribute to improvements in education and research in this area*” ^5^ (p. 4). And so our journal continues. In 1992, in its early days, the goals defined for the journal established a path for this project, from indexing to the peer review process ^6^.

Among editorials focused on editorial policy, research integrity ^7^ is a recurring topic, addressing issues about plagiarism ^8^ ^,^ ^9^ and authorship ^10^ ^,^ ^11^, for example. With the initiative of the Editors’ Forum of the Oswaldo Cruz Foundation (Fiocruz, acronym in Portuguese), in 2017, we became a member of the Committee on Publication Ethics (COPE), which aims to guide all actors involved in the publication process regarding ethical principles. The productivism required by scientist evaluation processes is directly related to issues of research integrity ^12^.

On the other hand, without ignoring their importance, the impact factors of journals clearly favor countries in the Global North ^13^. The themes cited by equally indexed journals are not necessarily relevant in the Global South. The classification and ranking of journals in these indexers and their influence on the selection process of professors and on the evaluation of postgraduate programs was the subject of a collective editorial ^14^ involving seven journals from Fiocruz. CSP became a signatory of the *San Francisco Declaration on Research Assessment* (DORA) ^15^, which indicates that less weight should be given to the journal classification based on these indicators. To support that, the CSP website does not highlight its impact factor.

More recent editorials have explained our priorities. *Innovation, Quality and Quantity: Choose Two* ^16^ and *More of the Same Epidemiology?* ^17^ summarize criteria for initial rejection of articles that are not selected for peer review: low relevance, little originality, and methodological inadequacy are the main reasons for such initial refusal. Guidelines for each CSP thematic subarea have been published in several editorials: epidemiological studies ^18^ ^,^ ^19^, literature reviews ^20^, evaluation of health services ^21^, research in digital environments ^22^, websurveys ^23^, book reviews ^24^. We also seek to make our job clear to authors and readers ^25^.

We can say that every topic, every event, every congress, every challenge in the field of Public Health has been addressed in an editorial at some point, as well as the directions of health policy, new (and old) diseases, inequalities, and inequities. The role of women in science and publishing is a frequent topic of our three Editors-in-Chief ^26^, but not new in the CSP: in the thematic issue published in 1991, the editorial said “*to conclude, we highlight that (with two honorable exceptions) all authors included here are female authors*” ^27^ (p. 134). Open science is also an increasingly present topic ^28^ ^,^ ^29^, associated with the challenges for a gradually more collective scientific practice ^30^.

Our initial intention in this editorial was to briefly highlight the CSP themes and positioning over these 40 years. But it was not possible to be brief. In these 40 years, the CSP editorials have reflected the most relevant topics studied, researched, and debated in the field. As we stated in another editorial: the CSP is a “common asset of Public Health” ^31^. In this sense, we seek to emphasize the contributions of the editorials, just like its equivalent in the press in general, which identify issues that permeate the different sections of the journal, expand the focus of the discussion, and reflect on our own role beyond the walls of academia.
